# Anti-Allergic Activity of a Platycodon Root Ethanol Extract

**DOI:** 10.3390/ijms11072746

**Published:** 2010-07-16

**Authors:** You-Chang Oh, Ok-Hwa Kang, Jang-Gi Choi, Young-Seob Lee, Obiang-Obounou Brice, Hyun Ju Jung, Seung-Heon Hong, Young-Mi Lee, Dong-Won Shin, Yeong-Shik Kim, Dong-Yeul Kwon

**Affiliations:** 1 College of Pharmacy and Wonkwang-Oriental Medicines Research Institute, Wonkwang University, Iksan 570-749, Korea; E-Mails: ulivuli@hanmail.net (Y.-C.O.); kangokhwa@hanmail.net (O.-H.K.); JJ0038@wku.ac.kr (J.-G.C.); st_andrea81@naver.com (Y.-S.L.); b_obiang@yahoo.com (O.-O.B.); hyun104@wku.ac.kr (H.J.J.); jooklim@wku.ac.kr (S.-H.H); ymlee@wku.ac.kr (Y.-M.L.); 2 Department of Oriental Medicine Resources, Sunchon National University, Jeonnam 540-742, Korea; E-Mail: sdw@sunchon.ac.kr; 3 Natural Products Research Institute College of Pharmacy, Seoul National University, Seoul 151-742, Korea; E-Mail: kims@snu.ac.kr

**Keywords:** antiallergy, Platycodon root, bone marrow-derived mast cell, prostaglandin D_2_, leukotriene C_4_, cyclooxygenase-2

## Abstract

*Platycodon grandiflorum* (Campanulaceae) is used as traditional medicine in Asian countries. In Korean traditional medicine, Platycodon root has been widely used since ancient times as a traditional drug to treat cold, cough and asthma. However, its effects on bone marrow-derived mast cell (BMMC)-mediated allergy and inflammation mechanisms remain unknown. In this study, the biological effect of Platycodon root ethanol extract (PE) was evaluated in BMMC after induction of allergic mediators by phorbol 12-myristate 13-acetate (PMA) plus calcium ionophore A23187 (A23187) stimulation. The effect of PE on the production of several allergic mediators, such as interleukin-6 (IL-6), prostaglandin D_2_ (PGD_2_), leukotriene C_4_ (LTC_4_), β-Hexosaminidase (β-Hex) and cyclooxygenase-2 (COX-2) protein, was investigated. The results demonstrate that PE inhibits PMA + A23187 induced production of IL-6, PGD_2_, LTC_4_, β-Hexosaminidase and COX-2 protein. Taken together, these results indicate that PE has the potential for use in the treatment of allergy.

## 1. Introduction

Platycodon root is the root of *Platycodon grandiflorum*, which belong to the family of Campanulaceae. In Asian countries, Platycodon root has been widely used since ancient times as a traditional drug. More specifically, it is often prescribed as a folk remedy and herbal medicine for coughs, sputum, asthma and tonsillitis disorder effects. Recent studies have shown that materials derived from Platycodon root are effective against asthma, have anti-inflammatory effects and act against fatty liver [1–3].

Mast cells are one of the most important cells for diseases related to allergic. When activated, mast cells release a number of biologically active molecules through various processes. Histamine, serotonin and serine proteases are released through exocytosis, lipid mediators such as eicosanoids are released through the activation of the cyclooxygenase-2 and 5-lipoxygenase (LOX) pathways and various cytokines are synthesized *de novo* [4–6]. They have long been implicated in the pathology and mortality of anaphylaxis and other allergic disorders by virtue of both their ability to be activated through FcɛRI-bound antigen-specific IgE and their concentration at surfaces that interface with the external environment [4]. Mast cells may also be activated by various cytokines through cytokine receptors. Activation through any of these cytokine receptors leads to release of a number of biologically active molecules, including histamine, serotonin, proteoglycans and neutral proteases. Among these molecules, histamine is one of the most important chemical mediators in the pathologic allergic reaction [7].

Interleukin-6 (IL-6) is an interleukin that acts as both a pro-inflammatory and anti-inflammatory cytokine. It is secreted by T cells and macrophages to stimulate immune response to trauma, especially burns or other tissue damage leading to inflammation [8,9]. Further, IL-6 modulates a variety of physiological events on the nervous system, endocrine system and bone metabolism [9,10].

Cyclooxygenase (COX) is a bi-functional enzyme that first catalyzes the addition of two molecules of oxygen to arachidonic acid to form the hydroperoxide prostaglandin G_2_ (PGG_2_), then reduces the hydroperoxide to the alcohol, PGH_2_, by a peroxidase activity [11]. It has two isoforms, COX-1 and COX-2. COX-1, which is constitutively expressed in most cells, is responsible for the production of prostaglandins that maintain homeostasis. In contrast, COX-2 is upregulated in inflammatory cells in response to an inflammatory stimulus and is responsible for the production of prostaglandins at the site of inflammation [12–15]. Therefore, there is an increasing interest in the use of COX-2 inhibitors.

In the present study, the ability of Platycodon root ethanol extract (PE) to suppress phorbol 12-myristate 13-acetate (PMA) and calcium ionophore A23187 (A23187)-induced IL-6, PGD_2_, LTC_4_ production and COX-2 protein expression in BMMC was investigated. The results show that PE has anti-allergic activity.

## 2. Results and Discussion

### 2.1. The Effects of PE on BMMC Viability

**Figure 1.**
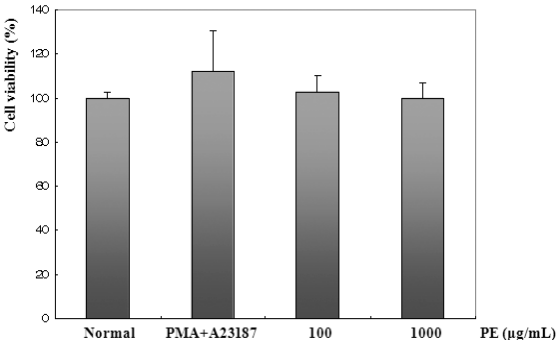
Effect of PE on BMMC viability. The cell viability of BMMC was assessed using an MTS assay following incubation with different doses (100 or 1000 μg/mL) of PE for 24 h. Values are the mean ± S.E. of duplicate determinations from three separate experiments.

Allergy is a disorder of the immune system often also referred to as atopy. Allergic reactions occur to normally harmless environmental substances known as allergens; these reactions are acquired, predictable, and rapid. Strictly, allergy is one of four forms of hypersensitivity and is called type I hypersensitivity. It is characterized by excessive activation of mast cells and basophils by a type of antibody known as IgE, resulting in an extreme inflammatory response [16]. The cytotoxic effect of PE was evaluated in BMMC by MTS assay (see Section 3). Figure 1 shows that treatment with PE at concentrations of up to 1000 μg/mL did not affect the viability of BMMC.

### 2.2. The Effects of PE on PMA and A23187-Induced IL-6 Secretion

**Figure 2.**
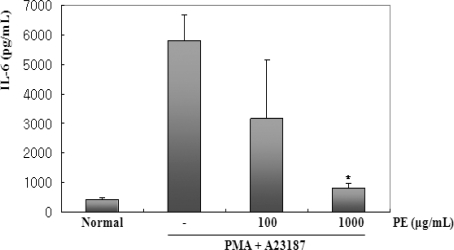
Effect of PE on the secretion of IL-6 in PMA + A23187-stimulated BMMC. BMMCs (1 × 10^6^ cells/mL) were pretreated with PE (100 or 1000 μg/mL) 30 min prior to stimulation with PMA (50 nM) and A23187 (1 μM). 6 h after PMA + A23187 stimulation, the IL-6 level of the supernatants were measured by ELISA. Statistical significance: *P < 0.05, as compared to the PMA + A23187 treated group. Significant differences between treated groups were determined using the Dunnett’s t-test. Values shown are the mean ± S.E. of duplicate determinations from three separate experiments.

IL-6 is released in a coordinated network and plays an important role in chronic disease. As such, the pattern of cytokine expression largely determines the nature and persistence of the inflammatory response [17]. IL-6 is a potent mediator of inflammatory processes, and a pleiotropic inflammatory cytokine produced by T cells, macrophages, monocytes, and synovial fibroblasts. To evaluate the effect of PE on the production of IL-6, we pretreated cells with PE (100 or 1000 μg/mL) before stimulation with PMA (50 nM) and A23187 (1 μM) for 6 h, followed by analysis using ELISA. As shown in Figure 2, the level of IL-6 considerably increased after stimulation with PMA plus A23187 in BMMC. Pretreatment of cells with PE (100 or 1000 μg/mL) inhibited these increases in a concentration-dependent and statistically significant manner.

### 2.3. The Effects of PE on PMA and A23187-Induced PGD_2_ Production

**Figure 3.**
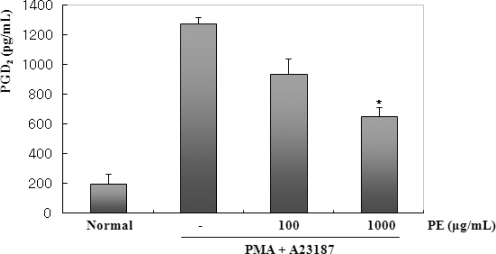
Effect of PE on the production of PGD_2_ in PMA + A23187-stimulated BMMC. BMCCs (1 × 10^6^ cells/mL) were pretreated with PE (100 or 1000 μg/mL) 30 min prior to stimulation with PMA (50 nM) + A23187 (1 μM). 2 h after PMA + A23187 stimulation, the PGD_2_ levels of the supernatants were measured by ELISA. Statistical significance: *P < 0.05, as compared to the PMA + A23187 treated group. Significant differences between treated groups were determined using the Dunnett’s t-test. Values shown are the mean ± S.E. of duplicate determinations from three separate experiments.

BMMC exhibit biphasic PGD_2_ biosynthetic responses over time, in addition to COX-1-dependent immediate and COX-2-dependent delayed responses [18]. The immediate PGD_2_ generation occurring within 2 h is associated with the coupling of COX-,1 and the delayed PGD_2_ generation, which occurs after several hours of culturing (between 2–10 h), is associated with the *de novo* induction and function of COX-2 after stimulation with particular stimuli [18]. This cell model also appears to be suitable for assessing the effect of 5-LOX inhibitors, since the immediate LTC_4_ generation elicited by the IgE-dependent or cytokine-initiated stimulus occurs in BMMC through 5-LOX [19]. Therefore, the BMMC system is useful for screening selective COX-1/COX-2 or 5-LOX and COX-2/5-LOX dual inhibitors from various sources [20,21]. After stimulation with PMA + A23187, cell-cultured mediums were harvested to measure the level of PGD_2_ production. PGD_2_ production was clearly detected 2 h after stimulation with PMA + A23187. PGD_2_ production also peaked in PMA (50 nM) + A23187 (1 μM). Next, an investigation of the inhibitory effect of PE on the PMA + A23187 induced PGD_2_ production in BMMC was performed. Figure 3 shows PE significantly inhibited PMA plus A23187-induced PGD_2_ production in dose-dependent manner. These results show that PE can inhibit PMA plus A23187 induced PGD_2_ production in BMMC.

### 2.4. The effects of PE on PMA and A23187-Induced LTC_4_ Production

**Figure 4.**
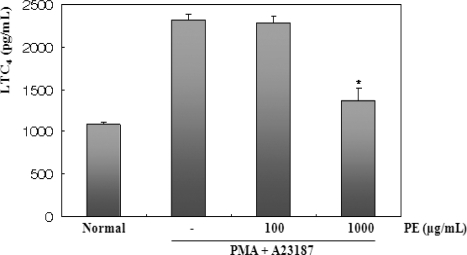
Effect of PE on the production of LTC_4_ in PMA + A23187-stimulated BMMC. BMMCs (1 × 10^6^ cells/mL) were pretreated with PE (100 or 1000 μg/mL) 30 min prior to stimulation with PMA (50 nM) + A23187 (1 μM). 15 min after PMA + A23187 stimulation, the LTC_4_ levels of the supernatants were measured by ELISA. Statistical significance: *P < 0.05, as compared to the PMA + A23187 treated group. Significant differences between treated groups were determined using the Dunnett’s t-test. Values shown are the mean ± S.E. of duplicate determinations from three separate experiments.

Arachidonic acid can also be converted to leukotrienes (LTs) by the action 5-lipoxygenase (LOX) in BMMC. The inhibition of 5-LOX is believed to be the ideal treatment for allergic diseases and asthma [22]. Therefore, the inhibitory activity of PE on the generation of LTC_4_ in the BMMC was examined. Figure 4 shows that the BMMC stimulated with PMA + A23187 for 15 min produced approximately 2.3 ng/mL LTC_4_, and preincubation of the BMMC with PE resulted in the dose-dependent suppression of this LTC_4_ biosynthesis. After stimulation with PMA plus A23187, cell-cultured mediums were harvested to measure the level of LTC_4_ production. LTC_4_ production was clearly detected 15 min after stimulation with PMA + A23187. LTC_4_ production also peaked in PMA (50 nM) plus A23187 (1 μM). Next, an investigation of the inhibitory effect of PE on the PMA and A23187 induced LTC_4_ production in BMMC was performed. Figure 4 shows PE significantly inhibited PMA plus A23187-induced LTC_4_ production in dose-dependent manner. These results show that PE can inhibit PMA + A23187 induced LTC_4_ production in BMMC.

### 2.5. The Effects of PE on PMA and A23187-Induced COX-2 Protein Expression

**Figure 5.**
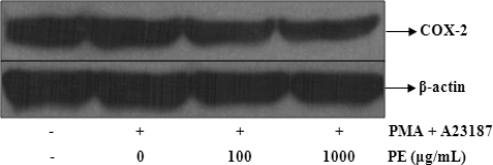
Effects of PE on PMA + A23187-induced COX-2 protein expressions in BMMC. BMMC were pretreated with the indicated concentrations of PE for 30 min prior to incubateion with PMA (50 nM) + A23187 (1 μM) for 6 h. Equal amounts of protein (20 μg) were then separated by SDS-polyacrylamide gel electrophoresis and immunoblotted with COX-2 antibodies. Equal loading of protein was verified by β-actin.

COX-2 is strongly induced in various activated cells. It has been reported that PGD_2_, which is the COX-2 metabolite released from activated mast cells, is also essential for the pathogenesis of eosinophilic airway [23]. The inhibitory effect of the PE on PGD_2_ production was examined to determine if it is a direct effect of the COX-2 protein or if this inhibition is mediated by some other mechanism. Western blot analysis was performed to determine if the inhibitory effect of PE on PGs were related to modulation of the COX-2 protein expression. As shown in Figure 5, the COX-2 protein was not detected in unstimulated BMMC. However, in response to PMA plus A23187, COX-2 protein was strongly expressed, and COX-2 protein expression was inhibited in a dose-dependent manner by PE.

### 2.6. The Inhibitory Effects of PE on PMA and A23187-Induced Degranulation

**Figure 6.**
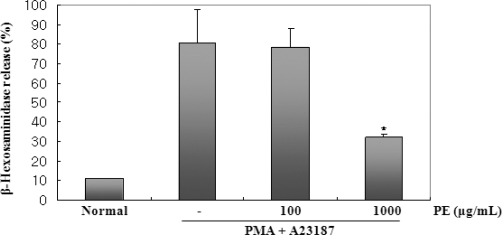
Effect of PE on the release of β-Hexosaminidase in PMA + A23187-stimulated BMMC. BMMC (1 × 10^6^ cells/mL) were pretreated with PE (100 or 1000 μg/mL) 30 min prior to stimulation with PMA (50 nM) + A23187 (1 μM). 90 min after PMA + A23187 stimulation, the β-Hex released into the supernatant cell lysate was measured by ELISA. The procedure is described in Materials and Methods in detail. All data are the arithmetic mean of triplicate determinations. Statistical significance: *P < 0.05, as compared to the PMA plus A23187 treated group. Significant differences between treated groups were determined using the Dunnett’s t-test. Values shown are the mean ± S.E. of duplicate determinations from three separate experiments.

When mast cells are activated by various stimuli, the release of histamine bears a close parallel to that of β-Hex, which is one degranulation marker [24]. An investigation of the effect of PE on the PMA p+ A23187-induced β-Hex release was performed using ELISA. As shown in Figure 6, pretreatment of BMMC with PE at concentrations of 100 or 1000 μg/mL reduced β-Hex production.

### 2.7. High Performance Liquid Chromatography (HPLC)

**Figure 7.**
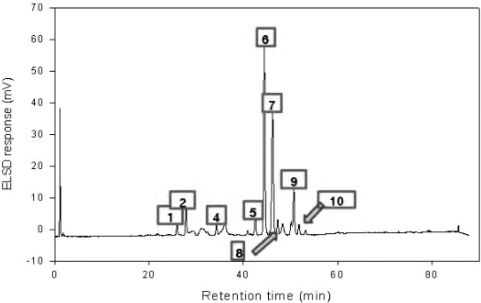
HPLC Chromatograms of PE.

HPLC analysis of saponins from Platycodon root was carried out on a Hitachi L-6200 instrument equipped with an evaporative light scattering detection (ELSD) system and SIL-9A auto injector (Shimadzu, Japan). A Zorbax SB-Aq C_18_ column (150 × 4.6 mm, 5 μm particle size) from Agilent Technologies (Palo Alto, CA, USA) was used for all separations. HPLC conditions were as follows: eluent A, water; eluent B, acetonitrile; gradient, 0–6 min (10–15% B), 6–50 min (15–25% B), 50–60 min (25–47.5% B), and then equilibrated with 10% B for 8 min at a flow of 1 mL/min. The ELSD was set to a probe temperature of 70 °C, a gain of 7 and the nebulizer gas nitrogen adjusted to 2.5 bar. As shown in Figure 7, peaks were assigned by comparing their retention times with that of each reference compound eluted in parallel with a series of mobile phase and by spiking sample with reference compounds. The standard solutions containing 2–400 μg/mL corresponding to test ranges of 10 saponins were prepared in triplicate and 50 μL of each sample was injected into the HPLC column for the construction of calibration curves and linear ranges. The calibration curves were plotted by the peak area *versus* concentration of each analyte. The linearity was evaluated by linear regression analysis calculated by the least square regression method.

The samples used in the previous experiment was purchased at a local herbal market and originated from China. In addition, the sample was extracted with 100% methanol [25]. In this experiment, the roots were collected in Korea and were extracted with 70% ethanol. These differences between the samples might affect the variation of saponin composition (Table 1).

## 3. Experimental Section

### 3.1. Plant Materials

Platycodon roots were collected from JinAn, Korea, in February, 2009. They were identified by Dr. D.Y. Kwon. A voucher specimen was deposited in the Laboratory of Herbalogy, College of Pharmacy, Wonkwang University, Iksan, Korea. The dried aerial parts of the Platycodon roots (1 kg) were cut into small pieces and extracted repeatedly with 70% ethanol. The solution was filtered and evaporated *in vacuo* to yield a powered extract (177.4 g).

### 3.2. Animals

Male Balb/cJ mice (20–30 g) were purchased from DAMUL, Korea. All animals were kept in a temperature controlled room under a 12 h light/12 h dark cycle. The animals were provided with free access to commercial solid food (SCF Co., Ltd. Korea) and water *ad libitum*. Mice were acclimatized for at least one week prior to beginning the experiments.

### 3.3. Drugs and Chemicals

RPMI 1640, penicillin and streptomycin and FBS (Fetal Bovine Serum) were obtained from Hyclone (Hyclone Labs Logan, UT). Bovine serum albumin (BSA), PMA and A23187 were purchased from Sigma (St. Louis, MO, USA). COX-2 and β-actin monoclonal antibodies and peroxidase conjugated secondary antibody were purchased from Santa Cruz Biotechnology Inc. (Santa Cruz, CA).

### 3.4. Preparation and Activation of Bone Marrow-Derived Mast Cells (BMMC)

Bone marrow mast cells from male Balb/cJ mice were cultured for up to 10 weeks in 50% enriched medium (RPMI 1640 containing 2 mM L-glutamine, 0.1 mM nonessential amino acids, antibiotics, and 10% fetal bovine serum) and 50% WEHI-3 cell-conditioned medium as a source of IL-3. After 3 weeks, >98% of the cells were BMMC.

### 3.5. MTS Assay for Cell Viability

The cell viability was examined by a 3-(4,5-dimethylthiazol-2-yl)-5-(3-carboxymethoxyphenyl)-2-(4-sulfophenyl)-2*H*-tetrazolium (MTS) assay. Briefly, BMMC were seeded at a density of 5 × 10^5^ cells/mL in 96 well plates (Nunc, Denmark). Each batch of cells included a non-treated group as control. PE (100 or 1000 μg/mL) was then added to each well, after which the plates were incubated for 24 h at 37 °C under 5% CO_2_. Next, MTS solutions (5 mg/mL) were added to each well. Next, the optical density was read at 490 nm. The cytotoxicity was then calculated by subtracting the ratio of the mean absorbance value for treated cells over the mean absorbance value for untreated cells from one.

### 3.6. Enzyme-Linked Immunosorbent Assay (ELISA)

Cells were seeded at a density of 1 × 10^6^ cells/mL in 24 well tissue culture plates and then pretreated with various concentrations of PE (100 or 1000 μg/mL) for 30 min prior to PMA (50 nM) plus A23187 (1 μM) stimulation. ELISA plates (Falcon, Becton Dickinson Labware) were then coated overnight at 4 °C with anti-mouse IL-6 antibody diluted in coating buffer (0.1 M carbonate, pH 9.5) and then washed three times with PBS containing 0.05% tween 20. Non-specific protein binding sites were then blocked by incubating the plates with assay diluent (PBS containing 10% FBS, pH 7.0) for at least 1 hour. Immediately following the incubation, each sample and IL-6 standard were added to the wells, after which the plates were incubated for 2 h. Next, working detector (biotinylated anti-mouse IL-6 monoclonal antibody and streptavidin-HRP reagent) was added to each well and the plates were incubated for an additional 1 h. Finally, substrate solution (tetramethylbenzidine: TMB) was added to the wells, after which the plates were then incubated in the dark for 30 min. Next, the reaction was stopped using stop solution (2NH_3_PO_4_) and the absorbance at 450 nm was then measured. All standards and samples were assayed in duplicate.

### 3.7. Determination of Prostaglandin D2 (PGD_2_) Levels

To measure the inhibitory activity of PE on COX-2, cells were suspended in enriched medium at 1 × 10^6^ cells/mL and preincubated with aspirin (10 g/mL) for 2 h to irreversibly inactivate any pre-existing COX-1. After washing, the BMMC were activated with PMA (50 nM) + A23187 (1 μM) at 37 °C for 2 h in the presence or absence of PE dissolved in PBS. All reactions were quenched by centrifugation at 890 *g* at 4 °C for 5 min. The supernatant and cell pellet were frozen immediately in liquid N_2_ and stored at −80 °C until needed for further analysis. Under these conditions, the COX-2-dependent phase of PGD_2_ generation reached 1.3 ng/10^6^ cells. The data are reported as the arithmetic mean of triplicate determinations.

### 3.8. Determination of Leukotriene C4 (LTC_4_) Levels

BMMC suspended in enriched medium at 1 × 10^6^ cells/mL were pretreated with PE for 30 min at 37 °C and stimulated with PMA (50 nM) + A23187 (1 μM). After 15 min of stimulation, the supernatants were isolated for further analysis by enzyme immunoassay (EIA). LTC_4_ was determined using an EIA kit (Cayman Chemical, Ann Arbor, MI, USA) according to the manufacturer’s instructions. Under the conditions employed, LTC_4_ reached 2.3 ng/10^6^ cells. All data are the arithmetic means of triplicate determinations.

### 3.9. Western Blot Analysis

After activation with PMA (50 nM) + A23187 (1 μM), BMMC were washed once with 10 mM phosphate buffer (pH 7.4) containing 150 mM NaCl (PBS) and lysed in PBS containing 0.1% SDS and 10 mM β-mercaptoethanol at 1 × 10^7^ cells/mL. The lysate (1 × 10^5^ cells equivalent) was applied to 10% SDS-polyacrylamide gels. After running the gel, the protein bands were blotted onto nitrocellulose membranes (Schleicher and Schull, Dassel, Germany) using a semi-dry blotter (MilliBlot-SDE system, Millipore, Bedford, MA, USA) according to the manufacturer’s instructions. Membranes were then washed once with 10 mM Tris-buffered saline (TBS, pH 7.2) containing 0.1% tween-20 (TBS-T), and then blocked for 1 h in TBS-T containing 3% skim milk. After washing the membranes with TBS-T, an antibody directed against COX-2 was added at a dilution of 1:5000 in TBS-T. After incubation for 2 h followed by washing three times, membranes were treated for 1 h with horseradish peroxide-conjugated goat anti-rabbit IgG (Santa Cruz, CA, USA) (diluted to 1:7000) in TBS-T. The protein bands were visualized using an enhanced chemiluminesence (ECL) system (Amersham Corp., Newark, NJ, USA).

### 3.10. Assay of β-Hexosaminidase Release

β-Hexosaminidase (β-Hex), a marker of mast cell degranulation, was quantitated by spectrophotometric analysis of p-nitrophenyl-2-acetamido-2-deoxy-β-*D*-glucopyranoside hydrolysis (Sigma, St. Louis, MO, USA). Briefly, after harvesting the supernatant, cells were lysed in the same volume of medium by three freeze/thaw cycles. Ten milliliters of the BMMC lysate or supernatant samples were mixed with 50 μL of β-Hex substrate solution (1.3 mg/mL *p*-nitrophenyl-2-acetamido-2-deoxy-β-*D*-glucopyranoside in 100 mM sodium citrate, pH 4.5) in the wells of 96-well plate and then incubated at 37 °C for 90 min. The reaction was stopped by adding 140 μL of 0.2 M glycine-NaOH (pH 10.7). Absorbance was measured at 410 nm in a microplate reader. The percentage of β-Hex released into the supernatant was calculated by the following formula: [S/(S + P)] × 100, where S and P are the β-Hex contents of the supernatant and cell pellet, respectively.

### 3.11. Chromatography

The chromatographic system consisted of a pump (Shimadzu LC-10AT VP) and a UV detector (Shimadzu LC-SPD-10A VP). A Shim-pack VP-ODS column (4.6 × 250 mm, Shimadzu) was used. An isocratic of CH_3_CN:CH_3_OH:H_2_O (11:11:8) was used as the mobile phase at a flow rate of 1.0 mL/min. The HPLC was monitored at 254 nm.

### 3.12. Statistical Analysis

The data from the experiments are presented as the mean ± S.E.M. The level of statistical significance was determined by analysis of variance (ANOVA) followed by Dunnett’s t-test for multiple comparisons. P values less than 0.05 were considered to be significant.

## 4. Conclusions

The conclusion of these investigations is that PE can exhibit inhibitory effects on PMA and A23187-induced IL-6, PGD_2,_ LTC_4_ production and COX-2 protein expression in bone marrow-derived mast cells. Considering the results, PE could represent a new drug treatment for various allergic diseases. Therefore, further studies are necessary to examine its clinical application.

## Figures and Tables

**Table 1.** Content of each compound.

**Number**	**Name**	**μg/mg**
1	Deapi-Platycoside E	1.4694
2	Platycoside E	1.6027
3	Deapi-Platycodin D_3_	ND
4	Platycodin D_3_	1.3450
5	Deapi-Platycodin D	1.6375
6	Platycodin D	5.6279
7	Polygalacin D	6.3629
8	3″-*O*-acetyl polygalacin D	2.3310
9	Platycodin A	2.7392
10	2″-*O*-acetyl polygalacin D	2.3579
